# Supplementary material for: WordCommentsAnalyzer: A windows software tool for qualitative research
Source: F1000Res. 2018 Sep 4;7:536. Originally published 2018 May 3. [Version 2] doi: 10.12688/f1000research.14819.2 (PMC6137411; doi:10.12688/f1000research.14819.2)
Supplement: Supplementary file 3 [file f1000research-7-17614-s0002.tgz › b2a79496-4ada-47c4-9682-a99ce867836d.zip!/Eight Iranian Health Journals/Yafteh _15.docx]

© Yafteh  . This work is licensed under CC BY 4.0 (https://creativecommons.org/licenses/by/4.0/).

Volume & Issue: دوره 16 - شماره 3

Title: بررسی تغییرات وزن، سطح سرمی قند خون ناشتا و استرس در دوره ی امتحانات پایان ترم نیمسال اول سال تحصیلی (91-90) دانشجویان دانشگاه علوم پزشکی لرستان

http://yafte.lums.ac.ir/article-1-1738-fa.html

مقدمه: یکی از مهم ترین موقعیت های تنش زا، امتحانات پایان ترم در دوره های دانشگاهی است. تنش و قرار گرفتن در شرایط تنش زا می تواند بر سطح سرمی قند خون و تغییرات وزن نیز مؤثر باشد. هدف از این مطالعه بررسی امکان استرس زا بودن امتحانات پایان ترم نیمسال اول سال تحصیلی (91-90)، تعیین میزان تأثیر گذاری قرار گرفتن در شرایط تنش زای امتحانات پایان ترم بر روی تغییرات وزن و سطح سرمی قند خون ناشتا (FBS) دو ماه قبل، یک ماه قبل و حین دوره امتحانات بر روی دانشجویان شاغل به تحصیل دانشگاه علوم پزشکی لرستان و بررسی رابطه بین این دو بوده است. مواد و روش‌ها: این مطالعه بر روی 226 نفر از دانشجویان شاغل به تحصیل در نیمسال اول سال تحصیلی (91-90) دانشگاه علوم پزشکی لرستان صورت گرفت و روش نمونه گیری به صورت تلفیقی از نمونه گیری طبقه ای و خوشه ای چند مرحله ای بود. وزن بدن با دقت 5/0 کیلوگرم و همچنین FBS توسط دستگاه گلوکومتر اندازه گیری شد. استرس توسط پرسشنامه بهداشت زندگی کودرون مورد بررسی قرار گرفت. یافته‌ها: قرار گرفتن در شرایط امتحانات پایان ترم تنش می تواند تنش زا باشد (001/0>P) در حالیکه در این شرایط تنش زا افزایش وزن در گروه های سنی کمتر به طور معناداری بیشتر بود (002/0=P). تغییرات میزان FBS با مقطع تحصیلی در ارتباط بود (034/0=P). میانگین وزن و FBS در مردان بالاتر بود (به ترتیب 001/0>P و 037/0=P). بحث و نتیجه‌گیری: بنابر نتایج این مطالعه تفاوت تغییرات وزن در دوران امتحانات پایان ترم دانشگاه علوم پزشکی لرستان از لحاظ آماری معنا دار نبود. این در حالی است که افزایش سطوح FBS در دوران امتحانات به طور معناداری دیده می شود.

Title: راهکارهای ارتقاء کیفیت مراقبتهای حین زایمان

http://yafte.lums.ac.ir/article-1-1739-fa.html

مقدمه: دسترسی به مراقبت‌های مناسب و با کیفیت یکی از مهم ترین راههای کاهش مرگ و میر مادر و نوزاد می‌باشد. هدف این مطالعه ارائه راهکاری ارتقاء کیفیت مراقبتهای حین زایمان استان لرستان در سال 1390 بود. بحث و نتیجه‌گیری: با توجه به نتایج بدست آمده، راهکارهایی همچون ایجاد انگیزه مالی، استفاده بیشتر از خدمات بخش خصوصی جهت کاهش بار کاری بخش دولتی و افزایش کیفیت و استفاده بیشتر از آموزش‌های ضمن خدمت جهت ارتقاء کیفیت خدمات توصیه می‌گردد. یافته‌ها: نتایج نشان دادکه مراقبت‌های مرحله اول زایمان دارای 5/54%، مرحله دوم 57% و مرحله سوم 66% تطابق با وضعیت مطلوب بودند و در هر سه مرحله کیفیت مراقبت ها متوسط ارزیابی گردید. بر اساس مصاحبه های انجام شده مسئولین دانشگاه علوم پزشکی لرستان راهکارهایی از جمله ایجاد انگیزه مالی برای ماماها و آموزش‌های ضمن خدمت را جهت ارتقاء کیفیت خدمات مناسب دانسته اند. مواد و روش‌ها: این پژوهش یک مطالعه ترکیبی (کمی، کیفی) است که طی آن کیفیت 200 مراقبت حین زایمان در مراکز درمانی استان لرستان مورد ارزیابی قرار گرفت. گردآوری اطلاعات از طریق ابزار محقق ساخته (چک لیست های تهیه شده بر اساس دستورالعمل های وزارت بهداشت) بود. جهت تجزیه و تحلیل اطلاعات از آمار توصیفی و نرم افزار SPSS استفاده گردید. در بخش کیفی نیز از طرق مصاحبه با ارائه دهندگان خدمت، مسئولین زایشگاه و مسئولین رده بالای دانشگاه علوم پزشکی (تصمیم گیرندگان) به بررسی راهکارهای ارتقاء کیفیت پرداخته شد.

Title: بررسی مقایسه فرآیند فتوکاتالیستی سیلیکا- اکسید روی با فرآیند فتوکاتالیستی اکسید روی در رنگبری و حذف COD

http://yafte.lums.ac.ir/article-1-1741-fa.html

مقدمه: یکی از معایب عمده فتوکاتالیست اکسید روی، عدم ثبات نوری این کاتالیزور در محلول های آبی به علت خوردگی نوری است که باعث کاهش عمده ای در فعالیت فتو کاتالیستی اکسید روی شده و کاربرد عملی آن را در پاکسازی محیط محدود می‌کند. در این مطالعه کارایی فرآیند فتوکاتالیستی سیلیکا-اکسید روی (UV/Silica-ZnO) در رنگبری و حذف COD با فرآیند فوتوکاتالیستی اکسید روی(UV/ZnO)مقایسه شد. بحث و نتیجه‌گیری: بر اساس نتایج بدست آمده بهترین درصد سیلیس در ترکیب با نانو ذرات اکسید روی 10 درصد بود بنابراین می توان از فرآیند UV/Silica-ZnO به عنوان یک روش موثر و کارآمد برای رنگبری از پساب های نساجی استفاده کرد. یافته‌ها: طیف XRD ، عدم وجود ناخالصی را در نانو ذرات تأیید می نماید. درصدهای مختلف سیلیس 5، 10 و 15 درصد در ترکیب با نانو ذرات اکسید روی مورد آزمایش قرار گرفت. نتایج نشان داد که میزان رنگبری و حذف COD در مدت تابش 90 دقیقه در فرآیند UV/Silica-ZnO با بارگذاری 10 درصد سیلیس به ترتیب برابر با 100 و 81 درصد و برای فرآیند فتوکاتالیستی UV/ZnO به ترتیب برابر با 66 و 44 درصد می باشد. مواد و روش‌ها: این مطالعه در مقیاس آزمایشگاهی انجام شده است. درصدهای مختلف نانو ذرات سیلیس جهت ترکیب کردن با نانو ذرات اکسید روی در فرایند UV/Silica-ZnO بر روی شیشه تثبیت شد. طیف پراکنش اشعه X (XRD) نانو ذرات اکسید روی به تنهایی و نانو ذرات اکسید روی ترکیب شده با سیلیس تهیه شد.

Title: بررسی اپیدمیولوژیک نارسایی مزمن و انتهایی کلیه و عوامل خطر آن در بیماران دیالیزی استان لرستان

http://yafte.lums.ac.ir/article-1-1742-fa.html

مقدمه: شیوع نارسایی انتهایی کلیه (ESRD) در دنیا در حال افزایش است. با توجه به اهمیت بالینی ESRD و فقدان اطلاعات کامل، این مطالعه با هدف بررسی اپیدمیولوژیک نارسایی مزمن و انتهایی کلیه در بین بیماران همودیالیزی استان لرستان انجام گردید. مواد و روش‌ها: این مطالعه مقطعی در فاصله زمستان 1390 تا زمستان سال 1391 در مراکز همودیالیز وابسته به دانشگاه علوم پزشکی لرستان انجام گردید. روش نمونه گیری بصورت سرشماری بود. اطلاعات بیماران با استفاده از پرسشنامه جمع آوری شد. در پایان، داده های جمع آوری شده با استفاده از نرم افزار SPSS و آمار توصیفی و آزمون آماری مجذور کای مورد تجزیه و تحلیل قرار گرفت. بحث و نتیجه‌گیری: ارتقاء روش های غربالگری و بخصوص ارجاع سریعتر بیماران در معرض خطر به نفرولوژیست، از ابتلای افراد به نارسایی انتهایی کلیه پیشگیری خواهد نمود یافته‌ها: در این مطالعه، تعداد بیمارانی که به علت نارسایی مزمن کلیه تحت همودیالیز قرار می‌گرفتند 318 نفر بود، که از این تعداد 182 نفر (2/57%) مرد و 136 نفر (8/42%) زن بودند. میانگین سنی بیماران 4/16±2/53 سال بود. علت نارسایی کلیه در 1/38% پرفشاری خون و در 2/19% و 4/27% موارد به ترتیب دیابت و علل ناشناخته بود. 97/5% بیماران مبتلا به یکی از عفونتهای ویروسی هپاتیتC، B یاHIV بودند. بین علل نارسایی مزمن کلیه در بیماران مورد مطالعه به تفکیک سن تفاوت آماری معنی داری دیده شد (002/0P= ).

Title: ارتباط بین فریتین سرم با سندرم متابولیک در بزرگسالان شهر خرم آباد

http://yafte.lums.ac.ir/article-1-1743-fa.html

مقدمه: شیوع سندرم متابولیک در حال افزایش است و شناسایی عوامل مرتبط با آن در بحث پیشگیری اهمیت زیادی دارد. هدف از این مطالعه تعیین ارتباط فریتین سرم و سندرم متابولیک در بزرگسالان شهر خرم آباد است. مواد و روش‌ها: در این مطالعه مقطعی مورد شاهدی تعداد 201 فرد غیر مبتلا به سندرم متابولیک (گروه شاهد) و177 فرد مبتلا به سندرم متابولیک (گروه مورد) مورد بررسی قرار گرفت. فریتین سرم با روش رادیوایمونواسی اندازه گیری شد. سندرم متابولیک بر اساس معیارهای ارائه شده از طرف برنامه ملی آموزش کلسترول پانل 3 درمان بزرگسالان تعریف شد. برای تجزیه و تحلیل داده ها از آزمونهای کوواریانس، کای اسکور و تی مستقل استفاده شد. بحث و نتیجه‌گیری: شیوع سندرم متابولیک در افرادی که سطح فریتین سرم بالایی داشتند نسبت به افراد با سطح فریتین پایین و طبیعی بیشتر بود. نتیجه گیری کلی اینکه ارتباط مستقیمی بین سطح فریتین و شیوع سندرم متابولیک وجود دارد. یافته‌ها: از 378 نفر شرکت کننده در مطالعه 9/74% زن و 2/71% متاهل بودند. 6/52% تحصیلات دیپلم یا کمتر داشتند. مهمترین شاخص هایی که با سندرم متابولیک ارتباط معنی داری داشتند. سن(001/0=P)، وضعیت تأهل(001/0=P)، میزان تحصیلات (001/0=P) و فریتین سرم (006/0=P) بودند. بین متغیرهای میزان درآمد، جنس، وضعیت مصرف سیگار با سندرم متابولیک ارتباط معنی داری دیده نشد.

Title: مقایسه توجه انتخابی بین بیماران افسرده، وسواسی، اضطرابی و افراد عادی

http://yafte.lums.ac.ir/article-1-1744-fa.html

مقدمه: با توجه به اینکه ما نمی توانیم همه اطلاعات را پردازش کنیم، باید بتوانیم اطلاعاتی را انتخاب کنیم که بیشترین اهمیت را برای ما دارند. ظاهراً بیماران مبتلا به اختلالات اضطرابی، به صورت انتخابی به محرک هایی که مرتبط با آن اختلال هاست توجه می کنند و از توجه به سایر اطلاعات غافل می مانند. به همین دلیل هدف این پژوهش بررسی مقایسه توجه انتخابی بین بیماران افسرده، وسواسی، اضطرابی و افراد عادی می باشد. بحث و نتیجه‌گیری: بر اساس نتایج به دست آمده و با توجه به اهمیت و کاربرد توجه انتخابی در زندگی روزمره توصیه می‌شود برای افزایش توجه انتخابی در این بیماران نهایت تلاش به کار گرفته شود. یافته‌ها: نتایج نشان داد که افراد افسرده در زمان واکنش سرعت عمل کندتری در مقایسه با سه گروه دیگر دارد. همچنین افراد اضطرابی و افراد وسواسی در مقایسه با افراد عادی، سرعت عمل کندتری دارند، اما سریعتر از افراد افسرده واکنش نشان می‌دهند. در زمینه تعداد خطا، نتایج نشان داد افراد افسرده در مقایسه با افراد عادی تعداد خطاهای بیشتری دارند، اما بین سایر گروهها در این زمینه تفاوت معناداری مشاهده نشد. مواد و روش‌ها: در این پژوهش از روش علی مقایسه ای استفاده شده است. جامعه آماری شامل تمام افراد عادی و تمام بیماران افسرده، اضطرابی و وسواس فکری - عملی می باشد که در سال 91 به کلینیک روانی دانشگاه علوم پزشکی لرستان مراجعه کرده اند و توسط روان پزشک تشخیص داده شده اند. برای بررسی هدف پژوهشی از بین جامعه آماری گروه نمونه ای به اندازه 120 نفر (30 افسرده، 30 اضطرابی، 30 وسواس فکری عملی و 30 نفر عادی) با استفاده از روش نمونه گیری در دسترس انتخاب شدند. جهت جمع آوری داده‌های این پژوهش، آزمون استروپ ( آزمون رنگ – واژه) به منظور اندازه گیری توجه انتخابی مورد استفاده قرار گرفت. در این تحقیق از نشانگرهای توصیفی (فراوانی، میانگین، انحراف استاندارد) و از آزمون استنباطی روش تحلیل چند متغیره و آزمون شفه استفاده شد

Title: تعیین میزان مایکوتوکسین زیرالنون در آرد نانوایی‌های شهر خرم آباد به روش کروماتوگرافی مایع با کارایی بالا

http://yafte.lums.ac.ir/article-1-1745-fa.html

مقدمه: زیرالنون یک مایکوتوکسین استروژنیک است که توسط چندین گونه از قارچ های فوزاریومی تولید می شود و به دلیل اثرات استروژنیک قوی و رخداد گسترده آن، مورد توجه خاصی قرار گرفته شده است. گندم و آرد آن یکی از مهمترین منابع غذایی انسان می‌باشند که ممکن است توسط زیرالنون آلوده شوند. بنابراین این مطالعه برای تعیین میزان آلودگی آرد گندم به زیرالنون انجام شد. بحث و نتیجه‌گیری: اگر چه میانگین غلظت زیرالنون در نمونه ها پایین تر از ماکزیمم سطح توصیه شده بوسیله کدکس بود، ولی وجود مقادیر زیرالنون در این نمونه ها، نیاز بهبود شرایط نگهداری به منظور کاهش سطح آلودگی زیرالنون در گندم را معلوم می دارد. مواد و روش‌ها: پس از استخراج زیرالنون از نمونه های آرد گندم با حلال استخراجی استونیتریل- آب ، مرحله خالص سازی سم بر پایه ستون های ایمونوافینیتی انجام گرفت. نمونه ها به کمک دستگاه HPLC با استفاده از ستون 18C با ابعاد mm 6/4×250 و اندازه ذرات µm 5 ، آشکارساز فلورسانس، فاز‌متحرک مخلوط استونیتریل و آب (3 به 2) با سرعت جریان ml/min 1 آنالیز شدند. یافته‌ها: نتایج مطالعه نشان دادکه در 5 درصد از نمونه های آرد، سطوح زیرالنون بالاتر از حد مجاز 200 میکروگرم بر کیلوگرم می‌باشد. میانگین غلظت زیرالنون در نمونه ها 54 میکروگرم بر کیلوگرم بود. حد تشخیص و حد کمی بودن توسط این روش به ترتیب 5/3 و 10 میکروگرم بر کیلوگرم بود.

Title: بررسی شیوع نگرش های مربوط به کنترل وزن و اختلالات خوردن و ارتباط آن با شاخص‌های تن‌سنجی در دختران نوجوان دانش‌آموز

http://yafte.lums.ac.ir/article-1-1746-fa.html

مقدمه: نوجوانی یکی از بحرانی ترین دوره های رشد انسان محسوب می شود. یکی از شایعترین بیماریهای تغذیه- روانی در این دوران، اختلال خوردن است که می تواند زمینه ساز مشکلات حاد و مزمن جسمی و روحی باشد. بنابراین مطالعه ای با هدف تعیین شیوع نگرش های مربوط به کنترل وزن و اختلالات خوردن و ارتباط آن با شاخص های تن سنجی در دختران نوجوان دانش آموز شیراز طراحی گردید. بحث و نتیجه‌گیری: به دلیل شیوع بالای اختلالات تغذیه‌ای در دختران نوجوان و همراه بودن سوء تغذیه از نوع چاقی و لاغری با این اختلالات، تصحیح این رفتار می تواند از اولویت های سلامت در این شهرستان باشد. یافته‌ها: داده‌ها نشان می‌دهد 80% جمعیت مورد مطالعه در معرض خطر اختلالات خوردن می‌باشند. به گونه‌ای که جمعیت دانش‌آموزان چاق، دارای اضافه وزن و لاغر در گروهی که دچار اختلالات نگرشی تغذیه‌ای بودند، به طور معنی‌داری بیشتر بود. مواد و روش‌ها: این مطالعه از نوع مقطعی توصیفی تحلیلی بود که با روش نمونه گیری تصادفی چند مرحله ای، 594 دانش آموز دختر دبیرستانی انتخاب گردیدند. با استفاده از پرسشنامه نگرش تغذیه‌ای، اطلاعات جمعیت جمع‌آوری شد. به منظور جمع‌آوری اطلاعات دموگرافیک و تن سنجی از پرسشنامه دیگری استفاده شد. با استفاده از نرم افزار SPSS و آزمون های T مستقل و کای اسکور اقدام به تجزیه و تحلیل اطلاعات گردید. 05/0< P به عنوان سطح معنی‌داری در نظر گرفته شد.

Title: بررسی اثر نانو ذره اکسید روی 20 نانومتری بر روی تغییرات آنزیمی و بافتی کبد موش باردار نژاد NMRI

http://yafte.lums.ac.ir/article-1-1747-fa.html

مقدمه: امروزه فن‌آوری نانو به سرعت در حال توسعه بوده و ممکن است اثرات قابل توجهی بر صنعت، جامعه و محیط زیست داشته باشد. در این مطالعه خصوصیات سمی نانو ذره اکسید روی بر بافت و آنزیم کبدی ALT در موش باردارNMRI بررسی شد. بحث و نتیجه‌گیری: نتایج ما نشان داد استفاده از غلظت های مختلف نانو ذره اکسید روی می‌تواند سبب اثر نامطلوب بر کبد با آسیب به هپاتوسیت و افزایش سطح آنزیم های کبدی شود. یافته‌ها: نتایج نشان داد که فعالیت آنزیم ALT با دریافت نانو ذره در غلظت های mg/kg50، 100، 150 و 200 در مقایسه با گروه کنترل در همه گروه ها در سطح معنی دار 05/0< P افزایش یافته است. بررسی هیستولوژی نشان داد که نانو ذرات اکسید روی سبب آسیب شدید کبد شد. آسیب به سلول های کبدی باعث پیشرفت نشت غشاء و آزاد شدن آنزیم به داخل خون می شود. مواد و روش‌ها: این مطالعه تجربی در شرایط استاندارد بر روی 25 سر موش سوری باردار با وزن متوسط 3 ±30 گرم انجام شد بطوریکه یک روز در میان به مدت 15 روز، دوز های مختلف نانو ذره اکسید روی را بصورت تزریق درون صفاقی دریافت کردند. سپس نمونه‌های خونی در روز هفده ام از موش‌های NMRI گرفته شد و بافت ها جهت بررسی هیستولوژی با بافر بوئن تثبیت شدند و با هماتوکسیلین و ائوزین رنگ-آمیزی شدند. نتایج حاصله توسط برنامه آماری SAS مورد تجزیه تحلیل قرار گرفتند.

Title: مطالعه اثر حفاظتی میکروامولسیون عصاره هیدروالکلی مریم نخودی بر روی سمیت کبدی ناشی از بروموبنزن در موش سفید کوچک

http://yafte.lums.ac.ir/article-1-1748-fa.html

مقدمه: کبد یک ارگان اصلی بدن است که ممکن است با مواد شیمیایی گوناگون، دارو ها و تعداد زیادی زنوبیوتیک نظیر بروموبنزن مورد مواجهه قرار گیرد. هدف از این مطالعه، ارزیابی اثر حفاظتی میکروامولسیون عصاره هیدروالکلی گیاه مریم نخودی بر روی سمیت کبدی ناشی از بروموبنزن می باشد. بحث و نتیجه‌گیری: نتایج نشان داد که میکروامولسیون عصاره هیدروالکلی گیاه مریم نخودی اثر محافظتی روی سمیت کبدی القاء شده توسط برومو بنزن را داراست یافته‌ها: نتایج این مطالعه نشان داد که جویز بروموبنزن سبب افزایش معنادار فعالیت آنزیم‌های کبدی می‌گردد. مصرف میکروامولسیون عصاره در دوزهای mg/kg100، 200 و 400 باعث کاهش معنی دار آنزیم های کبدی گردید. مشاهدات بافت شناسی نیز نتایج این مطالعه را تأیید کرد. مواد و روش‌ها: حیوانات به 8 گروه 10 تایی تقسیم شدند. گروه یک تا سه به ترتیب سرم فیزیولوژی، پایه میکروامولسیون و میکروامولسیون عصاره مریم نخودی با دوز mg/kg 400 به مدت 10 روز خوراکی دریافت کردند. گروه چهار پایه میکروامولسیون و گروه پنج تا هشت به ترتیب دوزهای mg/kg 50، 100، 200 و 400 از میکروامولسیون عصاره و سپس 1 ساعت پس از آخرین تجویز، ml/kg 36/0 بروموبنزن به صورت داخل صفاقی، دریافت کردند. 24 ساعت بعد، از حیوانات خونگیری به عمل آمد و فعالیت آنزیم های ALT، AST و ALP مورد اندازه گیری قرار گرفت. کبد حیوانات جهت مطالعات بافت شناسی جدا گردید.

Title: بررسی اثر کفش طبی با راکر پاشنه تا پنجه بر روی گشتاورهای وارد بر مفصل مچ پا و پارامترهای فضایی و زمانی راه رفتن در دانشجویان دختر

http://yafte.lums.ac.ir/article-1-1749-fa.html

مقدمه: راکرها یکی از رایج ترین اصلاحات خارجی کفش های طبی می باشند و در درمان مشکلات مچ پا به کار می روند. هدف از انجام این مطالعه مشخص کردن میزان دقیق تغییرات گشتاورهای وارد بر مفصل مچ و پارامترهای فضایی و زمانی راه رفتن هنگام استفاده از این راکر می باشد. بحث و نتیجه‌گیری: بر اساس یافته های موجود بزرگترین مزیت این کفش بدون تغییر ماندن سرعت حرکت و کاهش گشتاورهای وارد بر مفصل مچ پا در صفحه ساژیتال و در نتیجه کاهش بار وارد بر مفصل و تاندون آشیل می باشد، ولی افزایش گشتاورها در صفحه فرونتال سبب افزایش مقداری بی ثباتی در مفصل می شود. یافته‌ها: تفاوت میانگین گشتاورهای وارد بر مفصل مچ با کفش راکر در مقایسه با کفش معمولی در صفحه ساژیتال کاهش (002/0=P) و در صفحه فرونتال افزایش(007/0=P) یافت و این تفاوت ها از نظر آماری معنادار شد ولی تفاوت میانگین پارامترهای فضایی و زمانی راه رفتن از نظر آماری معنادار نشد. مواد و روش‌ها: مطالعه حاضر از نوع نیمه تجربی با نمونه گیری غیر احتمالی در دسترس بود و نمونه آماری 20 دانشجوی دختر، با الگوی راه رفتن طبیعی بودند. ابزار مورد استفاده دستگاه آنالیز حرکت وایکان 470 و صفحه نیرو کیستلر بود. برای هر فرد پارامترهای فضایی و زمانی و گشتاورهای وارد بر مفصل مچ پا ضمن راه رفتن با استفاده از هر دو نوع کفش محاسبه شد و نتایج حاصل با استفاده از آزمون t زوجی تحلیل شد.

Title: معرفی دختر مبتلا به سندرم استورج وبر همراه با بدشکلی استخوانی در ناحیه دهان- فک بالا- صورت

http://yafte.lums.ac.ir/article-1-1750-fa.html

مقدمه: سندرم استورج وبر جزء اختلالات پوستی عصبی نادر می باشد که فراوانی آن در جامعه 1 در 50000 مورد است و شامل گروهی از علایم و نشانه هاست که عبارتند از: خال صورت به رنگ قرمز آلبالویی، تشنج و ضعف یکطرفه در اندامها دربسیاری از موارد نیز ممکن است با عقب افتادگی ذهنی همراه باشد. بحث و نتیجه‌گیری: یکی از علائم رادیولوژیک ناشایع در سندرم استورج وبر بدشکلی استخوانی در ناحیه دهان- فک بالا - صورت است که تا کنون موارد اندکی گزارش شده است و در بیمار معرفی شده تمام این علائم یافت شده است. گزارش مورد: دختری 7 ساله با عقب ماندگی ذهنی که از بدو تولد خال صورت داشته و از 9 ماهگی مبتلا به تشنج های مکرر و آبریزش دهانی بوده و تا کنون تحت درمان دارویی قرارنگرفته است، به علت تشدید تشنج ها، سردرد، ضعف و بی حالی به اورژانس مراجعه کرده بود. در بررسی های انجام شده برای وی سندرم استورج وبر به همراه بدشکلی در استخوانهای دهان- فک بالا- صورت مطرح شد.
